# Seroprevalence of hepatitis B and C viruses among medical waste handlers at Gondar town Health institutions, Northwest Ethiopia

**DOI:** 10.1186/1756-0500-5-55

**Published:** 2012-01-22

**Authors:** Belay Anagaw, Yitayal Shiferaw, Berhanu Anagaw, Yeshambel Belyhun, Woldearegay Erku, Fantahun Biadgelegn, Beyene Moges, Agersew Alemu, Feleke Moges, Andargachew Mulu

**Affiliations:** 1 Department of Medical Microbiology, School of Biomedical and Laboratory Sciences, College of Medicine and Health Sciences, University of Gondar, Gondar, Ethiopia; 2 Department of Immunology and Molecular biology, School of Biomedical and Laboratory Sciences, College of Medicine and Health Sciences, University of Gondar, Gondar, Ethiopia; 3 Department of Statistics, Faculty of Natural and Computational Science, University of Gondar, Gondar, Ethiopia; 4 Department of Medical Parasitology, School of Biomedical and Laboratory Sciences, College of Medicine and Health Sciences, University of Gondar, Gondar, Ethiopia; 5 Aklilu Lemma Institute of Pathobiology, Addis Ababa University, Addis Ababa, Ethiopia; 6 Department of Medical Microbiology, College of Medicine and Health Sciences, Bahir Dar University, Bahir Dar, Ethiopia; 7 Institute of Virology, Faculty of Medicine, University of Leipzig, Leipzig, Germany

**Keywords:** Medical waste handlers, Hepatitis B virus, Hepatitis C virus, Gondar Ethiopia

## Abstract

**Background:**

Viral hepatitis is an inflammation of the liver due to viral infections and there are groups of viruses that affects the liver of which hepatitis B and C viruses are the causative agents of sever form of liver disease with high rate of mortality. Medical waste handlers who undergo collection, transportation, and disposal of medical wastes in the health institutions are at risk of exposure to acquire those infections which transmit mainly as a result of contaminated blood and other body fluids including injury with sharp instruments, splash to the eye or mucous membrane. This study aimed to determine the prevalence of hepatitis B and/or C viruses and associated risk factors among medical waste handlers.

**Results:**

A cross-sectional study was conducted from April, 2011 to June, 2011 in government health institutions at Gondar town. Socio-demographic and possible risk factors data from medical waste handlers were collected using pre-tested and well structured questionnaires. Venous bloods were collected and the serums were tested for hepatitis B surface antigen and anti-hepatitis C antibody using rapid Immunochromatography assay. Data was entered and analyzed using SPSS software package (version16). Chi-square and Fisher exact tests were used to assess risk of association. A p-value of < 0.05 was considered statistical significance.

A total of 100 medical waste handlers and 100 non-clinical waste handlers were examined for HBV and HCV viruses. HBV was detected in 6 (6.0%) and 1 (1.0%) and HCV in 1 (1.0%) and 0 (0.0%) of medical waste handlers and non-clinical waste handlers, respectively. Significant differences were observed in the detection rates of HBV (OR = 6.3; *X*^2 ^= 4.1; *P = 0.04*) and overall infection rate (HBV + HCV) (OR = 7.5; *X*^2 ^= 5.2; *P: 0.02*) in medical waste handlers when compared with non-clinical waste handlers. It was found that none of the observed risk factors significantly associated with rate of hepatitis infection compared to others.

**Conclusions:**

Prevalence of HBV and HCV were significantly higher in medical waste in relation to non-clinical waste handlers. There were poor waste management system which contributed for occurrence of higher degree of sharps injury and blood and body fluids splash.

## Background

Viral hepatitis is an inflammation of the liver due to viral infections and there are groups of viruses that affects the liver [1,2]. The most common types are hepatitis B virus (HBV) and hepatitis C virus (HCV) [2]. Viral hepatitis is a major health problem worldwide and cause acute and/or chronic hepatitis which can leads to the development of extensive liver scarring (cirrhosis), liver failure, liver cancer and death. Viral hepatitis is the tenth leading cause of death and the leading cause of liver cancer worldwide [3,4]. HBV and HCV can be ended with development of cirrhosis and liver cancer [5]. More than 500 million people worldwide are persistently infected with either of these two viruses thus presenting a major global health problem [6]. Because the two hepatotropic viruses share the same modes of transmission, co-infection with the two viruses is common, especially in areas with a high prevalence of HBV infection and among people at high risk for infection [7,8]. There are several million carriers worldwide which provide a huge reservoir for HBV and HCV. It may progress to chronic liver disease (CLD) including hepatocellular carcinoma (HCC) [9,10].

According to the estimate by World Health Organization (WHO), about two billion people worldwide have been infected with HBV and about 350 million people become chronic carriers and over one million people die each year as a result of acute fulminate liver disease or HBV induced cirrhosis and liver cancer [11,12]. The burden of HBV infection is highest in the developing world particularly in Asia and sub-Saharan Africa [13-15]. WHO estimated that the prevalence of HBV infection in Africa is on average more than 10% [16,17]. However, a study conducted in Addis Ababa showed that the mean prevalence of HBsAg was 6.1% [18].

WHO estimated also that approximately 170 million people are infected with HCV and about 130 million are carriers and three to four million persons are newly infected each year and more than 350,000 people estimated to die from hepatitis C-related liver diseases each year worldwide [19-21]. HCV infection in the world varies from 0.3 to 13% or more with the highest prevalence recorded in Central Africa and South-Eastern Asia [22,23]. However, a study in Addis Ababa showed that a 0.9% prevalence among population and 1.3% among adults over 15 years of age [24].

Both HBV and HCV are an important occupational hazard for medical waste handlers and chronically infected HBV and HCV carriers are able to transmit through contact with their blood and body fluids, which includes occupational exposure to their blood and body secretions. The current treatment for hepatitis B virus infection is not curable after the infection progress to chronic stage and very expensive for individuals in developing countries like Ethiopia. Thus early screening of People who are at risk including medical waste handlers is mandatory [25].

Generally, medical waste handlers who are working in collection, transportation, cleaning and disposal of medical wastes in health institutions have been consistently shown to have higher prevalence of HBV and HCV infection than non-clinical waste handlers that directly or indirectly have no contact with medical wastes [26,27]. The differences in hepatitis viruses infection rates may reflect disparities in the risk of exposure to infection [4]. Thus this study was conducted to determine the prevalence of HBV and HCV and associated risk factors among medical waste handlers working in health institutions of Gondar town.

## Methods

### Study design and area

A cross-sectional study was conducted into University of Gondar Teaching Hospital from April 2011 to June 2011. The study participants were recruited from six government health institutions found in Gondar town which is located in Northwest part of Ethiopia about738 km far from the capital city; Addis Ababa. Five of the six health institutions were health centers (Poly, Azezo, Teda, Maraki, and Welka) and the rest one was teaching and referral hospital (University of Gondar Teaching Hospital). These health institutions provide services to over 5 million inhabitants in the Northwest Ethiopia.

### Study subjects

One hundred medical waste handlers (MWHs) were included in the study (85 MWHs from University of Gondar Teaching Hospital and 15 MWHs from the five health centers). Participation in this study was voluntary based and all MWHs gave informed written consent. In order to see the type of waste handled has effect on magnitude of hepatitis virus infection, 100 non-clinical waste handlers (NCWHs) from the three campuses of University of Gondar (College of Medicine and Health Sciences, Tewodros and Maraki) were also included in the study. NCWHs were workers who clean class room, administrative offices, dining room, residence house, hospitals compound and handled non-clinical wastes generated from these areas like: paper, dust particles, leftover foods, dead plant tissues and others non-clinical wastes. Since it was difficult to match every property of non-clinical waste handlers with medical waste handlers, we selected all non-clinical waste handlers who were serve as cleaner in different Departments and Administrative Offices of the mentioned sites. Participants with previous vaccination for hepatitis virus and history of infection were excluded from the study. Those individuals identified positive during study were referred to University of Gondar Teaching Hospital's Internal Medicine Unit for management and further diagnosis.

### Sample collection and processing

#### Socio-demographic characteristics and exposure to risk factors

Pre-tested and structured questionnaire which consist of socio-demographic information, history of exposure to sharps and patient blood and body fluids in the past 1 year, HBV vaccination status, knowledge of infectious agents, provision of personal protective equipments (PPE) and others non-occupational risk factors was used. This information was collected with the help of trained senior nurse. In addition, interview (to explore attitudes towards the suitability and correct use of relevant personal protective equipment) and observation (use of PPE, PPE types, waste packaging, handling of wet wastes, sharps disposal procedures and waste management system) were also used as tool to generate data.

#### Blood sample collection and processing

After obtaining informed consent, 5 ml of venous blood was drawn under aseptic conditions from 100 MWHs and 100 NCWHs by experienced laboratory personnel and immediately put in a vacutainer tubes containing a clot activator. These tubes were numbered and processed at the time of collection. The blood samples taken from the individuals were centrifuged at 3000 revolution per minute (RPM) for at least 20 minutes at room temperature and the serum were separated and placed in eppendorf tubes. All serum samples collected from the five health centers were transported to University of Gondar Teaching Hospital Laboratory using cold box. Sera placed in eppendorf tubes were stored at -70°C until investigation was done.

#### Laboratory investigations

All the serum samples were tested for HBsAg and anti-HCV antibody using rapid diagnostic kits which work in the principle of immunochromatography (Human Gesellschaft fur Biochemical und Diagnostica mbH (HEXAGON), Germany, 2009). The sensitivity and specificity of the kits used as compared to the standard tests like ELISA was 100% and 99.8%, respectively [28,29]. Samples positive for either HBV or HCV were re-tested for second time by the same method. Samples repeatedly reactive for HBsAg or anti- HCV were considered positive. The subjects positive for HBV or HCV were referred and consulting to the internal medicine for further evaluation and treatment.

#### Quality control

Quality Control of both markers was checked based on the manufacturer kit instructions. The performance of the test kits were evaluated using known positive and negative control obtained from ELISA tested blood donor. Furthermore, formation of colored band to the control (C) line acts as a procedural control and serves to valid the results.

#### Data management and analysis

Data were entered and analyzed using SPSS software package (Version 16). Differences in proportions were evaluated by Pearson's *χ*^2 ^and Fisher exact tests; *p* < 0.05 was considered to be significant. Odds ratio was used as a measure of the strength of association.

#### Ethical considerations

The study was conducted after obtaining institutional ethical clearance from Research and Community Service Core Processor of the College. Information about the study was given to all waste handlers and was assured about the confidentiality, protection and anonymity of data. Written informed consent was obtained from voluntary study participants. Study participants that were identified positive were referred to health professionals for the management and for further investigations and follow-ups. In addition all information that was necessary for the management and further investigation was given to the respective health institutions as well as to the participants. Information obtained at any course of the study was kept confidential. Furthermore, during time of study on-site training was given to all medical waste handlers on how to handle, transport and dispose medical waste and about the possible infectious agents that can be encounter in medical waste handling.

## Results

### Socio-demographic characteristics

The socio-demographic characteristics of the medical waste handlers are presented in Table 1. The mean age was 34.7 ± 11.1 years (range from 18-57 years). The majority 96(96.0%) of the medical waste handlers were females (female to male ratio = 24:1) and 56(56.0%) of them had secondary school level or more educational status. The mean services years as medical waste handlers were 11 years.

**Table 1:** Socio-demographic characteristics of medical waste handlers (MWHs) and non-clinical waste handlers (NCWHs), at Gondar town Health institutions, 2011

Socio-demographic characteristics	Frequency (N = %)	
	
	MWHs (N = 100)	NCWHs (N = 100)
**Gender**		
**Male**	4(4.0)	3(3.0)
**Female**	96(96.0)	97(97.0)
**Age**		
**18-28**	37(37.0)	34(34.0)
**29-39**	22(22.0)	35(35.0)
**40-50**	35(35.0)	11(11.0)
**50-60**	6(6.0)	0(0.0)
**Marital status**		
**Single**	31(31.0)	49(49.0)
**Married**	47(47.0)	48(48.0)
**Divorced**	17(17.0)	2(2.0)
**widowed**	5(5.0)	0(0.0)
**Educational level**		
**Illiteracy**	2(2.0)	0(0.0)
**Writing & reading**	3(3.0)	1(1.0)
**Primary school**	39(39.0)	30(30.0)
**Secondary school**	42(42.0)	40(40.0)
**Certificate**	11(11.0)	26(26.0)
**Diploma & above**	3(3.0)	3(3.0)
**Year of services**		
**< 1**	22(22.0)	16(16.0)
**1-5**	31(31.0)	67(76.0)
**5-10**	8(8.0)	15(15.0)
**10-15**	6(6.0)	2(2.0)
**15+**	33(33.0)	0(0.0)
**Working institution**		
**GUH**	85(85.0)	25(25.0)a*
**HC**	15(15.0)	75(75.0)b *

*GUH *Gondar University Hospital, *HC *Health centres, a*: College of Medicine and Health Sciences; b *: Maraki and Tewodros Campus

### Seroprevalence of hepatitis infections

Among 100 MWHs and 100 NCWHs tested, HBV was detected in 6 (6.0%) and 1 (1.0%), and anti-HCV in 1 (1.0%) and 0 (0.0%), respectively [Table 2]. Significant differences were observed in the detection rates of HBV (OR = 6.3; *X*^2 ^= 4.1; *P = 0.04*) and total hepatitis viruses (HBV + HCV) (OR = 7.5; *X*^2 ^= 5.2; *P: 0.02*) in MWHs when compared with NCWHs.

**Table 2:** Distribution of HBV and HCV in medical waste handlers (MWHs) and non-clinical waste handlers (NCWHs) at Gondar town Health institutions, 2011

*Hepatitis infection*	*No. (%) Positive *		*OR*	*X^2^*	*P- *value
				
	MWHs(N = 100)	NCWHs(N = 100)			
**HBV**	6(6.0)	1(1.0)	6.3	4.1	0.04
**HCV**	1(1.0)	0(0.0)	Ud	1.4	0.24
**Total**	7(7.0)	1(1.0)	7.5	5.2	0.02

*N *total sample size

*Ud *undefined, *OR *Odd Ratio, *X^2 ^*Chi-square

### Socio-demographic characteristics and their association with hepatitis viruses

Analysis of socio-demographic characteristics and their association with hepatitis viruses was indicated in Table 3. Though, it was not statistically significant higher proportion of HBV & HCV were detected in female (RR = 4.0; 95%CI = 0.62-25; *P = 0.25*), married (RR = 1.5; 95%CI = 0.35-6.6; *P = *0.70), mean age ≤ 35 years (RR = 1.18; 95%CI = 0.28-5.0; *P = 1.00*), secondary school education level (RR = 0.95; 95%CI = 0.23-4.04; *P = 1.00) *medical waste handlers [Table 3]. Both HBV and HCV positive MWHs 7(8.2%) were from Gondar University Hospital working in different wards, OPD, laundry, and laboratory sections; although statistically non-significant (RR = 0.92; 95%CI = 0.86-0.98; *P = *0.59).

**Table 3:** Socio-demographic characteristics of MWHs and their association with hepatitis viruses infection at Gondar town Health institutions, 2011

Characteristics	Total (N = 100)	HBV or HCV positive (n = 7)	Negative for both HBV and HCV (n = 93)	RR(Risk ratio 95% CI)	P-value
Gender					
Male	4(4%)	1(14%)	3(3%)	1.0	
Female	96(96%)	6(86%)	90(96%)	4.0(0.62-25)	0.25*
Age in year					
≤ 35	53(53%)	4(57%)	49(53%)	1.0	
> 35	47(47%)	3(43%)	44(47%)	1.18(0.28-5.0)	1.00
Marital status					
Married	47(47%)	4(57%)	43(46%)	1.0	
Unmarried	53(53%)	3(43%)	50(54%)	1.5(0.35-6.4)	0.70
Educational level					
Elementary and below	44(44%)	3(43%)	41(44%)	1.0	
Secondary and above	56(56%)	4(57%)	52(56%)	0.95(0.23-4.04)	1.00
Economic status					
Low	54(54%)	4(57%)	50(54%)	1.0	
Medium	46(46%)	3(43%)	43(46%)	1.14	1.00
Years of services					
< 15 years	67(76%)	6(86%)	61(66%)	1.0	
> 15 years	33(33%)	1(14%)	32(34%)	2.95(0.37-23.5)	0.42
Institution					
Hospital	85(85%)	7(100%)	78(84%)	0.92(0.86-0.98)	0.59
Health centers	15(15%)	0(0%)	15(16%)	1.0	

*Fisher exact test

*N *total sample size of MWHs

*n *number of positive or negative for hepatitis virus

### Possible risk factors and their association with hepatitis virus infection

MWHs exposed to various work related as well as non-work related risk factors such as needle stick and other sharp injuries, body fluids splash in eye, nose, and mucus membrane, and others were presented in Table 4. It was found that none of the observed risk factors significantly associated with rate of hepatitis infection compared to others. All MWHs had no history of contact with cuts or open wounds, family liver diseases, tattooing, pre and post-exposure prophylactics related idea, immunized against HBV, and also had not been tested for HBV and HCV before and after employment [Table 4].

**Table 4:** Possible risk factors and their association with hepatitis virus infection at Gondar town Health institutions, 2011

*Risk factors*	*HBV or HCV positive (n = 7)*	*Negative for both HBV and HCV (n = 93)*	*Total (N = 100)*	*RR(Risk ratio 95% CI)*	*P-value*
**Blood transfusion**					
Yes	1(14%)	2(2%)	3(3%)	1.0	
No	6(86%)	91(98%)	97(97%)	5.4(0.91-31.9)	0.19*
**Needle stick & others sharp object injury**					
Yes	5(71%)	38(41%)	43(43%)	1.0	
No	2(29%)	55(59%)	57(57%)	3.31(0.67-16.3)	0.14
**Splash of body fluid to the eye, nose, & mucus membrane**					
Yes	4(57%)	40(43%)	44(44%)	1.0	
No	3(43%)	53(57%)	56(56%)	1.7(0.40-7.2)	0.69
**Tooth extractions**					
Yes	3(43%)	32(34%)	35(35%)	1.0	
No	4(57%)	61(66%)	65(65%)	1.39(0.33-5.9)	0.69

*Fisher exact test

*N *total sample size of MWHs

*n *number of positive or negative for hepatitis virus

### Measures taken when needle stick injury and splash to eye, nose, or mucus membrane in medical waste handlers

In the present study it was also reported that when needle stick injury and splash of blood and other body fluids occurred to the eye, nose or mucus membrane, 55.0% said they consulted health professionals (though gone to Voluntary Counseling and Testing (VCT) centers for fear of HIV/AIDS not for viral hepatitis), 36.0% had disinfected it and 9.0% had done nothing. Similarly, of those who had splashed body fluids to the eyes and mucus membranes, 22.0% had consulted health professionals and attended VCT centers, 56% had washed with clean waters and 22.0% had done nothing [Table 5].

**Table 5:** Measures taken when needle stick injury and splash to eye, nose, or mucus membrane in medical waste handlers at Gondar town Health institutions, 2011

Measures taken when	Frequency (N = %)		
	
	HBV or HCV positive (n = 7)	Negative for both HBV and HCV (n = 93)	Total (N = 100)
**Needle stick & others injury**			
**Consult health professionals**	6(86%)	49(53%)	55(55.0%)
**Disinfected it**	1(14%)	35(38%)	36(36.0%)
**No action taken**	0(0.0%)	9(9%)	9(9.0%)
**Splash to eye or mucus membrane**			
**Consult health professionals**	2(29%)	20(21%)	22(22%)
**Disinfected it**	4(57%)	52(56%)	56(56%)
**No action taken**	1(14%)	21(23%)	22(22%)

*N *total sample size of MWHs

*n *number of positive or negative for hepatitis virus

### Status of participants to universal precaution practices and awareness

None of the MWHs was immunized against hepatitis B virus and aware about viral hepatitis transmission via sexual contact, sharing special tools (i.e. tooth brushes, shaving razor, etc.) and intravenous drug abuse. However, all MWHs were aware that medical waste contains harmful germs. Regarding the training, 35(35.0%) took training, though had no difference in awareness with those who were not trained on viral hepatitis infection. Only 2(5.7%) of those trained had infection, although this was not statistically significant (RR = 2.3; 95%CI 0.54-9.6; *P *= 0.42).

Regarding the intention to used personal protection equipment, all MWHs were aware that wearing protective clothes is very important to protect them from infections associated with handling medical waste. However, none of the MWHs used eye goggle, and wore overalls.

Among those that used thick disposable gloves, only 5 (6.4%) of MWHs were infected by hepatitis which was not statistically different from those not used thick disposable gloves (RR = 0.71; 95%CI 0.15-3.4; *P = *0.65). Similarly, 1(2.2%) of MWHs were infected among those who used face masks while handling wastes but this not statistically significant (RR = 0.19; 95%CI 0.02-1.6; *P = *0.12). Seventy-three (73.0%) used protective gown, even if it was not appropriate gown (plastic apron) which were regularly supplied. Among this, 6(8.2%) were infected by hepatitis but which was not statistically significant (RR = 2.2; 95%CI 0.28-17.6; *P = *0.67). Moreover, 11(11.0%) of them used to wear boots while in working time, even if not statistically significant (RR = 1.3; 95%CI 1.02-1.15; *P = *1.00) [Table 6].

**Table 6:** Status of universal precaution follows up among medical waste handlers at Gondar town Health institutions, 2011

*Types of PPE used*	*HBV or HCV positive (n = 7)*	*Negative for both HBV and HCV (n = 93)*	*Total (N = 100)*	*RR(Risk ratio 95% CI)*	*P-value*
**Thick disposable gloves**					
Yes	5(71%)	73(78%)	78(78%)	1.0	
No	2(29%)	20(22%)	22(22%)	0.71(0.15-3.4)	0.65*
**Face masks**					
Yes	1(14%)	45(48%)	46(46%)	1.0	
No	6(86%)	48(52%)	54(54%)	0.19(0.02-1.6)	0.12
**Boots**					
Yes	0(0%)	11(12%)	11(11%)	1.0	
No	7(7%)	82(88%)	89(89%)	1.3(1.02-1.15)	1.00
**Protective gown**					
Yes	6(86%)	67(72%)	73(73%)	1.0	
No	1(14%)	26(28%)	27(27%)	2.2(0.28-17.6)	0.67
**Training**					
Yes	4(57%)	33(35%)	37(37%)	1.0	
No	3(43%)	60(66%)	63(63%)	2.3(0.54-9.6)	0.42

*Fisher exact test

*N *total sample size of MWHs

*n *number of positive or negative for hepatitis virus

## Discussion

### HBV and HCV seroprevalence

In Ethiopia data about HBV and HCV among medical waste handlers are lacking. Hence the present study tried to provide the seroprevalence of HBV and HCV in medical waste handlers and some possible risk factors observed in this group of people. The present study has found that 7.0% of MWHs had either HBV (6.0%) or HCV (1.0%). This rate is significantly higher (OR = 7.5; *X*^2 ^= 5.2; *P: 0.02*) than that in NCWHs (1.0%).

### Comparison with similar studies

Lower and higher prevalence rates were also detected as compared to similar study populations in different parts of the world. Lower prevalence found in Tripoli, Libya with reported rate of 2.3% HBV; whereas 2.7% HCV positivity which was slightly higher [26]; and prevalence of 1.59% also reported in Palestinian medical waste handlers [30]. A higher prevalence rate was reported from Turkey due to injury with various blunt and penetrating objects among housekeepers of whom >70% had been working in a University Hospital [27]. It is worth mentioning that the injuries among housekeepers in Turkey occurred while collecting waste material in the hospital [27]. In Rio de Janeiro, Brazil, a higher rate of 12.9% and 14.2% for HBV in hospital and municipal waste collection workers were reported, respectively [31]. This difference might be due to large sample size, immunization status and methodology used.

The present study showed that high prevalence rate of HBsAg (6.0%) positivity as compared to male (1.0%). Totally opposite result reported in Libyan's study where none of female medical waste handlers were positive compared to 2.9% HBsAg positivity in male [26]. This discrepancy might be due to the fact that the majority of the study participants in Libyan's study were male (four times the number of female participants). However; in our case almost all of the study participants were female (24:1). Regarding impact of educational status on the risk of acquisition of hepatitis viruses, no significant difference was observed between those who had secondary or above (4.0%) and those with primary or less school education (3.0%). Comparable result was obtained in Libya [26].

### Comparison with related domestic studies

In Ethiopia, previous population-based surveys had reported medium to high endemicity of viral hepatitis HBV and HCV infection [20,32,33]. Although direct comparison is difficult because of methodological and sample size differences, the prevalence obtained in the present study was 6.0% for HBsAg and for 1.0% anti-HCV appears to be in line with a 7% HBsAg and 1.3% ant-HCV prevalence reported previous among the general population in Addis Ababa [18,24] and a 7.3% HBsAg and 1.3% anti-HCV among antenatal attendees of Gondar health center [34]. It is also consistent with the rate in women attending antenatal clinics in Addis Ababa (5%) HBsAg [35]; to a previous estimate of HBV infection rate in the voluntary counseling and testing (VCT) clients and known HIV-positive cases of St Paul's General Specialized Hospital, Addis Ababa 4.7% HBsAg [36] and also the study in Gondar University Teaching Hospital among blood donors HBsAg 4.7% and anti-HCV 1.3% [37]. However, the present finding was comparable with a studies conducted to pregnant women in Jimma (South west part of Ethiopia) 3.7% HBsAg [38]; with Armed Forces General Hospital, prevalence rate was 9.02% HBsAg [33], but street dwellers in Gondar city resulted 10.9% HBsAg prevalence [39]. This difference might be due to the methodology used, large sample size and the study participants were all health care workers and administrative staff and there are highly vulnerable groups for sexual transmitted disease and sharing of sharp materials potential to hepatitis infections, respectively.

### Sharps injuries & splash exposures

Blood Born Virus (BBV) infection may follow needle or sharps injury, contamination of pre-existing skin lesions or splash inoculation to the eyes, nose or mucous membranes [40]. The present study reported that, 25.0% of the medical waste handlers were found to have needle stick or sharp injuries while handling medical waste. This finding was inconsistent with the studies revealed in Nigeria where needle stick injuries among healthcare workers were the commonest forms of exposure to HBV infections [41]. In Italy, needle sticks constituted the most common source of exposure (58.4%), followed by non-intact skin and mucous membrane contamination (22.7% and 11.2% respectively), and cuts (7.7%) [42].

Occurrence analysis revealed 60% of MWHs experienced multiple sharp injuries in similar sites i.e., on fingers and palms during collection, transporting, decanting and disposal of sharps and the rest took place on legs and different body parts (data not presented here). Even though it was difficult to tell here the exact figure for the share of different types of reason for the occurrence of ships injuries due to recall problem, incorrect and inadequate closure of sharp containers and sharp carelessly discarded into waste sack intended only for soft wastes were responsible for these injuries.

Similarly, 44.0% of the study participants were also exposed to blood or others body fluid splash in mouth, nose and eye. This finding was comparable to the study done in UK and China [40,43]. Participants also indicated carrying of overfilled waste bags, compression of overfilled waste bags/sacks and sharp-edged tearing waste bags/sacks were the major reason for generation and occurrence of blood and body fluids splash.

### Personal Protective Equipment (PPE) usage

All medical waste handlers knew that PPE can protect them from infection, though 55% did not use PPE regularly that had 9.1% hepatitis viruses positivity compared to those individual who regularly used PPE 4.4% and more than 85% did not wear boots while performing their duties. This may be the result of lack of training, as < 40% of MWHs were trained to handle medical waste, and shortage of supply since the majority of MWHs complained about shortage of PPE. This was comparable with the study in Libya [26] and Palestinian [30] which found that the level of occupational safety is below standard requirements, as protective equipment and clothing were not available for most workers and only < 20% and 37.2% of the workers were trained in handling medical waste, respectively.

The incidents of sharp injury and splash among MWHs were unacceptably high. It may be prevented though the use of puncture-resistant gloves, poly cotton trousers, penetration-proof masks and protective glasses. Use of PPE is not the only solution for preventing the occurrence of sharp injury and splash. Effective segregation of wastes at source and the correct use of waste containers provide the most effective safeguards [44].

### Waste management system

This study discovered that segregation of all waste materials was not conducted according to definite rules and standards. Sometimes hazardous waste was stored in the same containers as the household waste, and no control measures existed for the management of these waste materials. None of the health institution provided strong plastic bags for medical waste segregation and most of the time they used thin plastic bags that can easily tear. None used color coded plastic bags. The same kind and color of household waste bags were used for medical waste. They used any color available in the market, which was normally black, for both general and medical waste materials. In these health institutions, most departments disposed sharp in reusable plastic cans, except in the laboratory where they sometimes used carton sharp boxes when available. Similar situation was observed study conducted in Palestine [30].

It is important to collect and properly contain syringes and needles at the point of use and should seal before it is completely full. After closing and sealing, sharp containers must not be opened, emptied, reused, or sold [45]. In the same manner free flowing liquid waste should contain in leak-proof, rigid durable containers [43]. Red or orange bags are usually used for infectious waste. The containers should also be marked with the universal symbol for biological hazards. Infectious wastes should be contained from the point of origin to the point at which they are not longer infectious [46].

When the waste is to be moved about for treatment or storage, special handling or packaging may be necessary to keep bags intact and to ensure containment of the waste. Single-bagged waste and containers of sharp and liquid should be placed within a rigid or semi rigid container such as a bucket, box, or carton lined with plastic bags. Containers should be covered with lids during transportation and storage. When handling or transporting plastic bags of infectious waste, care should be taken to prevent tearing the bags. Infectious waste should not be compacted before treatment. This process could damage the packaging and disperse the contents, or it could interfere with the effectiveness of treatment. Outside the hospital, infectious waste should be transported in closed, leak proof dumpsters or trucks [46].

### Training and supply

Healthcare management should provide education and training to waste generators, handlers, collectors, transporters, and waste treatment facility operators. Handlers must be trained and equipped to undertake the handling, internal transport, spill management and storage requirements for the different types of wastes arising at the facility. The purpose of education and training is to minimize the risk of injury associated with waste handling and facilitate efficient waste management [47]. However, in the studied health institutions the training offered to waste handlers were inadequate and lack regularity. Some of MWHs received training which last for short duration and most did not obtain any training at the time of employment.

The management of each health institution is responsible for the purchasing of sharps and others waste containers. In all studied health institutions because of financial problems there was shortage of safety boxes or proper waste bin. So they used overfilled safety boxes to save these materials. Again shortage and ill fitted size personal protective devices were common in the hospitals. As indicated, few amounts of money were allocated every time for waste management sectors. This financial deficit created problems for continuous availability of waste management equipments and PPE for waste handlers. This could aggravate the existing problems and continuous occurrence of accidents in waste disposal sectors.

### Documentation of waste handling errors, injury and exposure

Since there were no well established waste management policies and accident management sections in the studied health institutions, almost all waste handling errors and injuries were not documented. Documentation of waste handling errors, injury and exposure in work place contribute a lot for preventing the re-occurrence of similar cases and a source of information for policy maker to develop and improve rule and regulation.

In most of the cases MWHs took their own measures either alone or with consultation of Infectious Disease Clinic to reduce the chance of infection following injuries. According to article 92 of the Labor Law of Federal democratic Republic of Ethiopia (issued 2003, numbered 377), it is the responsibility of employers to take appropriate steps to ensure that workers are properly instructed and notified concerning the hazards of their respective occupations and the precautions necessary to avoid accident and injury to health; register employment accident and occupational diseases and notify the labor inspection of same and ensure that the work place and premises do not cause danger to the health and safety of the workers. However, this study indicates that the relevant articles of the law and the actual activities are not complied with.

### Immunization

In Ethiopia universal infant HBV immunization started in 2007. However, there is no universal availability of the vaccine for adult population and it may be the reason that none of the medical waste handlers in the present study were immunized. Concerning immunization status present study showed inferior result 0.0% compared to studies takes place in Turkey 27.5% [27], Libya 21% [26] and UK 21% [40]. This might be due to the availability of free HBV immunization to risk groups, intensive periodic educational program and implementation of universal precautions which were absent in our study areas.

## Conclusion

The overall prevalence rates of hepatitis B and C viruses were higher among MWHs in the government health institutions of Gondar town compared to NCWHs. Workers' information about and awareness of viral hepatitis infection were inadequate. Furthermore, there was no rule and regulation for waste managements, medical wastes were collected, transported and disposed with inappropriate containers, inadequate supply of PPE and no organized sections responsible for documentation of waste handling errors and related injuries and exposures. Therefore, this group of workers is confronted with higher risk than other non exposed groups.

If hospital waste is not managed properly, it proves to be harmful to the environment. It poses a threat not only to the employees working in the hospital, but also to the people surrounding that area. This is true for studied health institutions. As result, these health institutions should promptly adopt procedures and policies for proper management of waste generated. Emphasis should be given to all stages from generation to final treatment and disposal of the waste through the establishment of source separation, sound sanitary collection and storage of medical wastes; ensuring transportation by approved packaging; treatment of wastes by safe and environmentally-sound methods, disposal of final residues in confined and carefully-designed sites.

Accident reporting helps to prevent the re-occurrence of similar cases. As result MWHs should encouraged to report by providing the necessary and expected help, advice and sympathy. Efforts also required introducing free vaccination at least to risk groups. Proper medical waste management is not a one time and section activities rather requires: cooperation of each and every members of the hospitals, continued education of all health care workers on proper disposal of equipment and commitment of higher officials for adequate budget allocation for waste disposal sectors.

## Competing interests

The authors declare that they have no competing interests.

## Authors' contributions

BA was the primary researcher, conceived the study, designed, participated in data collection, conducted data analysis, drafted and finalized the manuscript for publication. YS, YB and BM assisted in data collection and reviewed the initial and final drafts of the manuscript. BA, FM and WE interpreted the results, and reviewed the initial and final drafts of the manuscript. All authors read and approved the final manuscript.
